# Crowdsourcing scoring of immunohistochemistry images: Evaluating Performance of the Crowd and an Automated Computational Method

**DOI:** 10.1038/srep43286

**Published:** 2017-02-23

**Authors:** Humayun Irshad, Eun-Yeong Oh, Daniel Schmolze, Liza M. Quintana, Laura Collins, Rulla M. Tamimi, Andrew H. Beck

**Affiliations:** 1 Beth Israel Deaconess Medical Center, Harvard Medical School, Department of Pathology, Boston, 02115, USA; 2 Kaiser-Permanente, Mid-Atlantic Group, Rockville, MD, USA; 3 City of Hope National Medical Center, Duarte, CA, USA; 4 Harvard School of Public Health, Department of Epidemiology, Boston, 02115, USA; 5 Channing Division of Network Medicine, Brigham and Women’s Hospital, Boston, 02115, USA

## Abstract

The assessment of protein expression in immunohistochemistry (IHC) images provides important diagnostic, prognostic and predictive information for guiding cancer diagnosis and therapy. Manual scoring of IHC images represents a logistical challenge, as the process is labor intensive and time consuming. Since the last decade, computational methods have been developed to enable the application of quantitative methods for the analysis and interpretation of protein expression in IHC images. These methods have not yet replaced manual scoring for the assessment of IHC in the majority of diagnostic laboratories and in many large-scale research studies. An alternative approach is crowdsourcing the quantification of IHC images to an undefined crowd. The aim of this study is to quantify IHC images for labeling of ER status with two different crowdsourcing approaches, image-labeling and nuclei-labeling, and compare their performance with automated methods. Crowdsourcing- derived scores obtained greater concordance with the pathologist interpretations for both image-labeling and nuclei-labeling tasks (83% and 87%), as compared to the pathologist concordance achieved by the automated method (81%) on 5,338 TMA images from 1,853 breast cancer patients. This analysis shows that crowdsourcing the scoring of protein expression in IHC images is a promising new approach for large scale cancer molecular pathology studies.

Immunohistochemistry (IHC) is widely used for measuring the presence and location of protein expression in tissues. The assessment of protein expression by IHC provides important diagnostic, prognostic and predictive information for guiding cancer diagnosis and therapy. In the research setting, IHC is frequently evaluated using tissue microarray (TMA) technology, in which small cores of tissue from hundreds of patients are arrayed on a glass slide, enabling the efficient evaluation of biomarker expression across large numbers of patients.

The manual pathological scoring of large numbers of TMAs represents a logistical challenge, as the process is labor intensive and time consuming. Over the past decade, computational methods have been developed to enable the application of quantitative methods for the analysis and interpretation of IHC-stained histopathological images^1^,^2^. While some automated methods have shown high levels of accuracy for IHC markers^3^,^4^,^5^,^6^, automated analysis has not yet replaced manual scoring for the assessment of IHC in the majority of diagnostic pathology laboratories and in many large-scale research studies.

In this study, we evaluate the use of crowdsourcing to outsource the task of scoring IHC labeled TMAs to a large crowd of users not previously trained in pathology. Over the last decade, crowdsourcing has been used in a wide range of domains, including astronomy^7^, zoology^8^,^9^,^10^, medical microbiology^11^, and neuroscience^12^,^13^,^14^, to achieve tasks that required large-scale human labeling, which would be difficult or impossible to achieve effectively using only computational methods or domain experts.

In a pilot study, we explored the use of crowdsourcing for rapidly obtaining annotations for two core tasks in computational pathology: nucleus detection and segmentation^15^. This study concluded that aggregating multiple annotations from a crowd to obtain a consensus annotation could be used effectively to generate large-scale human annotated datasets for nuclei detection and segmentation in histopathological images. Crowdsourcing has also recently been evaluated for immunohistochemistry studies. Della Mea *et al*. crowdsourced 13 IHC images for detection of positive and negative nuclei and reported 0.95 Spearman correlation between pathologist and crowdsourced positivity percentages^16^. Recently, the http://CellSlider.netCellSlider project by Cancer Research UK provided an online interface for members of the general public to score IHC stained TMA images, and they reported high levels of concordance of crowdsourced scores obtained from non-experts and the scores of trained pathologists^17^.

The purpose of the present study is two-fold. First, we aim to evaluate the performance of crowdsourcing vs. an automated method for scoring protein expression in IHC stained TMA images. Second, we aim to evaluate the time, cost, and accuracy of two different approaches to crowdsourcing the IHC task (image-level labels vs. nucleus-level labels).

## Methods

### Dataset

The Nurses’ Health Study (NHS) cohort was established in 1976 when 121,701 female US registered nurses ages 30 to 55 responded to a mail questionnaire that inquired about risk factors for breast cancer^18^. Every two years, women are sent a questionnaire and asked whether breast cancer has been diagnosed, and if so, the date of diagnosis. All women with reported breast cancers (or the next of kin if deceased) are contacted for permission to review their medical records so as to confirm the diagnosis. Pathology reports are also reviewed to obtain information on ER and PR status. Informed consent was obtained from each participant. This study was approved by the Committee on the Use of Human Subjects in Research at Brigham and Women’s Hospital and all experiments were performed in accordance with the relevant guidelines and regulations.

This study used IHC-stained TMA images of breast cancer tissue from the NHS. The dataset consists of 5,338 scanned images of TMA cores, which were immunostained for estrogen receptor (ER) and scanned using Aperio Slide Scanner at 20× magnification. The size of each TMA core is 0.6 mm. After scanning, we extracted an image for each TMA core that contained only the tissue regions. The sizes of these TMA images are variable and depend on the amount of tissue present from each core. The average image size is 828 × 848 pixels. These images are derived from 1853 patients, each of whom contributed 1–3 TMA images, with more than half of the patients contributing 3 TMA images. All study images were scored by an expert breast pathologist, using three labels (negative = 0, low positive = 1 and positive = 2)^19^.

### Crowdsourcing Platform

We employed the CrowdFlower platform to design both crowdsourcing applications (image labeling and nuclei labeling). CrowdFlower is a crowdsourcing platform that works with over 50 labor channel partners to enable access to a network of more than 5 million contributors worldwide. This platform offers a number of features to improve the likelihood of obtaining high-quality work from contributors. In CrowdFlower, the job designer creates a job in the form of tasks, which are served to contributors for labeling. Each task is a collection of one or more images sampled from the data set. The job designer creates test questions (test images which have been previously labeled by pathologists) that are used for dual purposes: qualification of contributors during quiz mode and monitoring of contributors during judgment mode. Contributors must maintain a defined level of accuracy on the test questions to be permitted to complete the job. In addition, the job designer specifies the payment per task and the number of labels desired per image. After job completion, CrowdFlower provides a list of labels (annotations) for all the images. Additional information on the CrowdFlower platform is available at www.crowdflower.com.

### Job Design and Crowdsourcing Applications

Each crowdsourcing job has two modes: quiz mode and judgment mode. Quiz mode occurs at the beginning of a job. In quiz mode, there is only one task and the task consists of 5 test question images. In judgment mode, there are a number of tasks and each task consists of 4 actual images and one test image which is presented to the contributor in the same manner as the unlabeled images such that the contributor is unaware if he/she is annotating an unlabeled image or test image. Each contributor must qualify during quiz mode to enter in judgment mode and can remain in judgment mode as long as his/her accuracy on test questions is above a threshold level. For ensuring high quality of labels, we defined five parameters which may influence labeling performance.

- The first is test question minimum accuracy that ensures each contributor must maintain minimum 60% accuracy on test questions throughout the job completion.
- The second is minimum time per task that ensures each contributor must spend a minimum of 10 seconds to complete one task.
- The third is maximum number of judgments per contributor that enable more contributors to participate in the job. In our jobs, we defined maximum number of judgment per contributor 500 judgments.
- The fourth is a minimum number of images (20) for the contributor to review in work mode prior to computing a trust score for each contributor and prior to filtering contributors based on their trust score.
- The fifth is the number of labels to collect per image. We collect three labels per image for both jobs.

**Figure 1:**
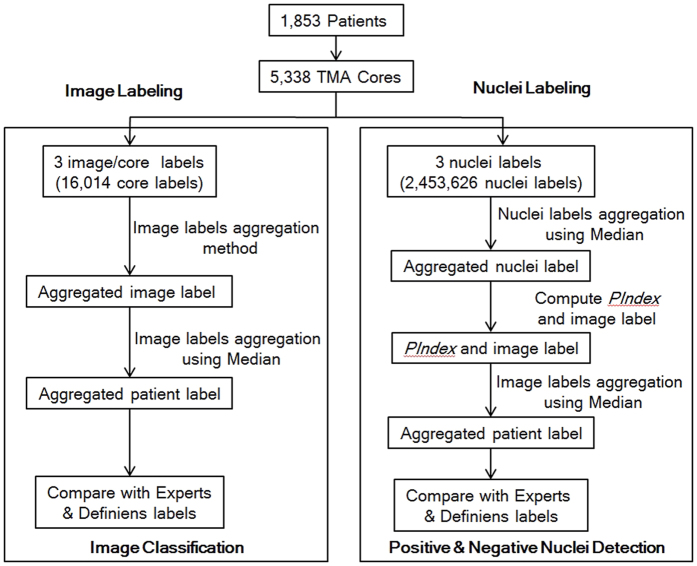
Crowdsourcing work flow for Image Labeling and Nuclei Labeling.

Our study includes two types of labeling jobs: image labeling and nuclei labeling. Figure 1 illustrates the flow chart of both crowdsourcing jobs. Each job contains instructions, which provide examples of expert-derived labels and guidance to assist the contributor in learning the process of labeling.

#### Image Labeling

**Figure 2:**
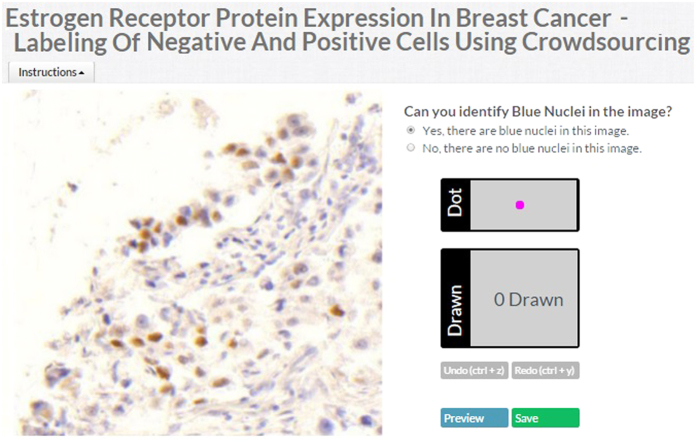
Crowdsourcing application interface for Image Labeling. The screenshot illustrates the interface for selecting the image class label.

In the image labeling job, each contributor estimates the percentage of cancer nuclei stained brown (positive) and blue (negative) in the image and then selects the image label (score) depending on given criteria: if percentage of brown nuclei is less than 1% then image label is A (negative protein expression), if percentage is between 1% and 10%, then image label is B (low positive protein expression), if percentage is between 10% and 50% then image label is C (positive protein expression) and if percentage is more than 50% then image label is D (high positive protein expression). The total pool of test question images used in both quiz and judgment modes are 250, which are labeled by pathologists. Figure 2 shows the interface for image labeling.

#### Nuclei Labeling

**Figure 3:**
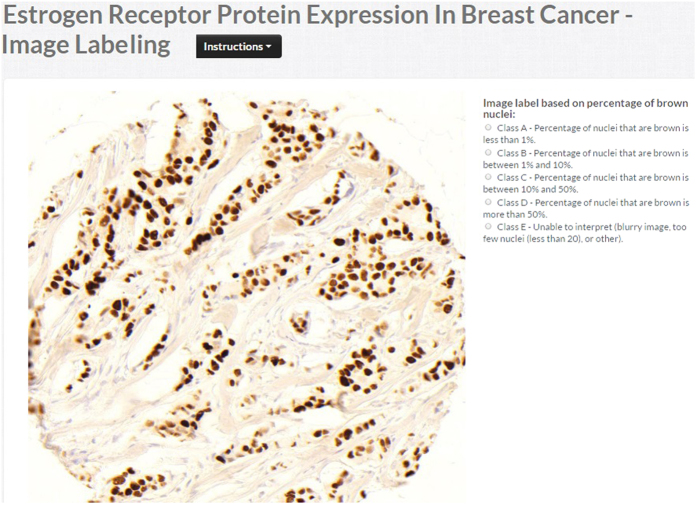
Crowdsourcing application interface for Nuclei Labeling. The screenshot illustrates the interface for labeling the positive and negative nuclei separately.

In the nuclei labeling job, we ask contributors to detect positive and negative nuclei in the image. Figure 3 shows the interface for nuclei labeling. In the nuclei labeling job, we first ask contributors to identify the presence of nuclei in the image (yes/no). If they do identify the presence of nuclei, then we ask the contributor to label the nuclei using a dot operator (by clicking at the center of each nucleus). At completion of job, CrowdFlower provides the position of positive and negative nuclei in the images. For each image, we collect positive and negative nuclei from three different contributors. The total pool of test question images are use in both quiz and judgment modes for nuclei labeling are 100 images, which are labeled by pathologists. After counting number of positive and negative nuclei, we compute the positivity index 
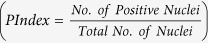
. From positivity index, we compute the image labels (A, B and C) according to following image labeling criteria:


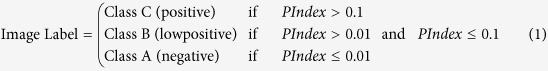


### Aggregation Methods for Image Labeling Problem

We calculated the aggregated label for each image using four different methods: maximum crowd votes (CV), maximum crowd trust scores (CT), maximum weighted crowd votes (*ω*CV) and maximum weighted crowd trust scores (*ω*CT). CV is computed by summing the votes for each label and selecting the label with the maximum number of votes as the aggregated label. CT is computed by summing the contributor trust score (CT) for each label and selecting the label with the maximum trust score as the aggregated label. For *ω*CV and *ω*CT methods, we multiply the class weights with crowd votes for each label and crowd trust scores for each label, respectively.


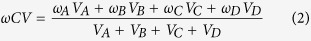



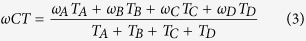


where *V*_*A*_, *V*_*B*_, *V*_*C*_ and *V*_*D*_ are crowd votes from each class labels; *T*_*A*_, *T*_*B*_, *T*_*C*_ and *T*_*D*_ are sum of crowd trust scores for each class labels; and *ω*_*A*_, *ω*_*B*_, *ω*_*C*_ and *ω*_*D*_ are class weights. We calculated the class weights by taking the mean of lower and upper boundary of the class. For class A, lower boundary is 0 and upper boundary is 0.01, the weight of class A is 0.005. For class B, lower boundary is 0.01 and upper boundary is 0.1, the weight of class B is 0.05. For class C, lower boundary is 0.1 and upper boundary is 0.5, the weight of class C is 0.3. For class D, lower boundary is 0.5 and upper boundary is 1, the weight of class D is 0.75. The selected aggregated label is the label whose class bounds contain the weighted crowd vote or weighted crowd trust score.

### Sensitivity Analysis for Different Combinations of Crowd Size

To estimate the number of crowd labels required to generate optimal aggregated crowd label, we performed a sensitivity analysis of aggregated labels using different combination of crowd sizes. For this pilot study, we collected 10 crowd labels for each image, and we computed the aggregated label of each image using different combination of crowd sizes (1 to 10), according to Algorithm 1.


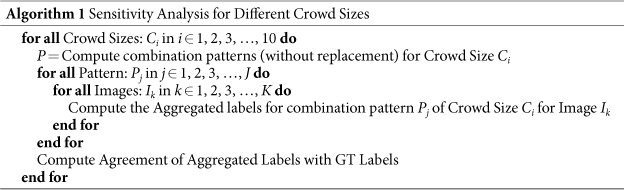


### Definiens Tissue Studio Pipeline for Image Labeling

There are three major steps in a Definiens Tissue Studio pipeline. (i) Epithelial-Stromal Classification: One representative TMA slide was chosen from all the slides in the analysis, and 12 tissue cores were then chosen from that slide for training of epithelial-stroma classifier. Epithelial and stromal regions were then labeled by the user in an iterative training process wherein the user can supervise the learning of the Definiens epithelial-stromal classifier. (ii) Nucleus detection: Nuclei were detected using hematoxylin and IHC marker thresholding only in epithelial regions. In our data set, hematoxylin was used to stain nuclei blue or purple and the IHC marker (3,3′-Diaminobenzidine) stained brown to indicate the presence of the peptide of interest. Negative nuclei were detected using a hematoxylin threshold, and positive nuclei were detected using an IHC marker threshold. The hematoxylin threshold was set so as to include the lightest negative epithelial nucleus while still excluding non-epithelial, non-nuclear tissue. The IHC marker threshold was set in a similar manner; so as to include the lightest stain positive nucleus while excluding non-epithelial, non-nuclear tissue, or background. (iii) Positivity Index Calculation: Once epithelial nuclei have been classified as positive or negative, positivity index was computed as 
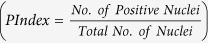
. Later, *PIndex* was converted into image labels (A, B and C) according to image labeling criteria (mentioned in section Nuclei Labeling section).

### Performance Measures

We explored different performance measures to evaluate inter-observer reliability of scores. For measuring the inter-observer reliability, we measured percent agreement (*A*_*g*_) or accuracy, which is calculated as the number of agreed labels divided by total number of labels, Kappa (*κ*) which measures the agreement among observers adjusted for the possibility of by chance agreement, Spearman correlation (*ρ*) which measures the mean of bivariate Spearman’s rank correlations between observers for inter-observer reliability, and intra-class correlation (ICC). For image classification, we used confusion matrix and *A*_*g*_ performance measures for comparing different methods of label aggregation and the automated method.

## Results

### Image labeling on 380 TMA cores - A pilot study

We designed a pilot study to test the crowd sourcing application for IHC image labeling and to assess the improvement in crowdsourcing performance as we increase the numbers of aggregated instances per image. In the pilot study, we collected 10 crowdsourced labels for 380 images. We also collected three pathologist labels for each of these 380 images using the same crowd sourcing interface.

**Figure 4:**
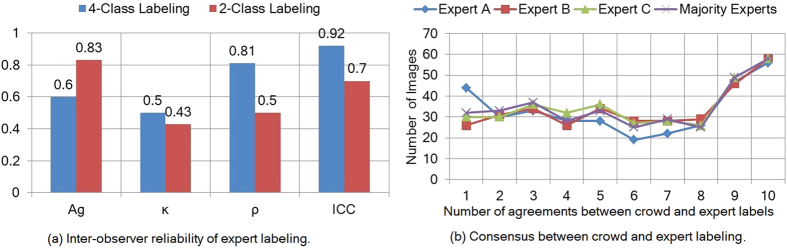
Inter-observer reliability of pathologist labels and agreement with crowd labels.

We assessed inter-observer reliability among pathologists using 4-class labeling as well as 2-class labeling as shown in Fig. 4(a). For 2-class labeling, we merged all positive classes (B, C and D) into a single positive class (B). We observed Kappa values of 0.43 and 0.5 for 2-class and 4-class labeling, respectively, indicating moderate inter-pathologist agreement in IHC interpretation.

**Figure 5:**
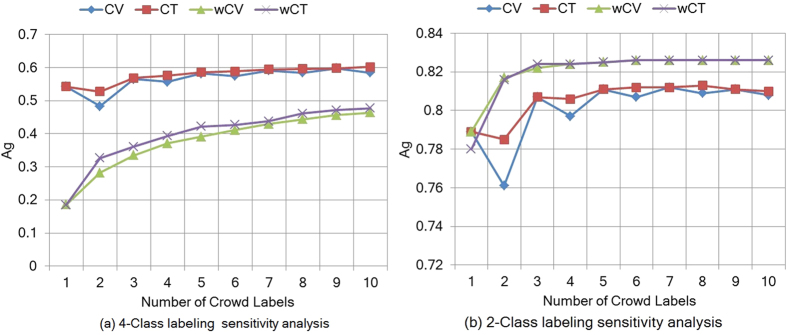
Sensitivity analysis of crowd labels in the pilot study. The analysis supports using 3 crowdsourced labels per image.

For 380 images, we obtained 10 crowd labels per image and compared these scores with the pathologist scores as shown in Fig. 4(b). We found a wide range of agreement on images between the crowd and the pathologist scores, with a median level of agreement of 6/10 and 15% of images showing 10/10 agreement. For a range of crowd labels per image (ranging from 1 to 10), we computed aggregated labels and assessed the agreement of the consensus score with the pathologist score for each number of crowd labels as reported in Fig. 5. The *A*_*g*_ is not significantly improved after crowd size 3 for 4-class and 2-class image labeling problem.

### Image labeling on 5338 TMA cores

Based on the results of the sensitivity analysis, we collected 3 crowd labels for each image for the image and nucleus labeling crowdsourcing jobs for the main study. The image and nuclei labeling work flow is illustrated in Fig. 1. For 4-class image labeling, we collected 3 labels for each TMA image using CrowdFlower as shown in Fig. 2. In total, 16,014 image labels were collected for 5,338 images. Aggregated image labels were computed with four aggregation methods (CV, CT, *ω*CV and *ω*CT). The aggregated label at patient level was computed by taking the median of all aggregated image labels belonging to that patient. Pathologists labeled these images using 3-class labeling: negative (A), low positive (B) and positive (C). Since we obtained 4-class labeling from the crowd, to compare the crowd labels with pathologist labels, we merged crowd class D into crowd class C. The CV aggregation method reported higher *A*_*g*_ and Spearman *ρ* than other aggregation methods for 3-class labeling as reported in Table 1. For 2-class labeling, we merged all positive classes (B and C) into a single positive class (B) for both crowd and pathologist aggregated labels. The CV aggregation method outperformed as compared to other aggregation method for *A*_*g*_ and *ρ* as reported in Table 1.

### Nuclei Labeling on 5338 TMA cores

For the nuclei labeling job, we collected 3 nuclei labels for all 5,338 TMA images. Total number of nuclei labels was 2,453,646. The aggregated number of positive and negative nuclei was calculated for each image as the median number of positive and negative nuclei labeled by the crowd. Then, we computed *PIndex* for each image. *PIndex* was converted into image labels (A, B and C) according to criteria (mentioned in Nuclei Labeling section).

Lastly, we computed the aggregated patient label by taking the median of the all the image labels belonging to that patient and compared with the pathologist labels as reported in Table 1. We also performed 2-class labeling by merging all positive classes into a single positive class for both crowd and pathologist labels. The *A*_*g*_ and *ρ* are 0.77 and 0.68 for 3-class labeling and 0.87 and 0.63 for 2-class labeling, respectively.

In order to compare with an automated method, we developed an image processing pipeline in Definiens Tissue Studio. This pipeline detected positive and negative nuclei in TMA images and computed the *PIndex*. The crowdsourcing *PIndex* was correlated with the Definiens *PIndex (ρ* is 0.75). However, considering pathologist labels as ground truth, both types of crowdsourcing jobs (image and nuclei labeling) resulted in higher *A*_*g*_ and *ρ* than Definiens for both 3-class labeling and 2-class labeling.

The Crowd showed significantly improved performance on test questions for the nuclei labeling job as compared with the image labeling task as reported in Table 2. This finding supports the overall higher level of accuracy seen with the nuclei labeling approach as compared with the image labeling approach.

Number of true and false labeled images are reported in the form of a confusion matrix in Table 3. Definiens pipeline for image labeling misclassified most negative and low positive cases into positive cases, which may be due to under-segmentation of those epithelium regions having low or no ER expression resulting in less detection of negative nuclei. In case of crowdsourced image labeling, most negative and low positive cases are labeled positive suggesting that most contributors underestimate the count of negative cells for estimating the image labels. Another possible reason of under-estimating negative cells could be low contrast and difficulty in detecting negative cells as compared to positive cells. In Crowdsourced based Nuclei Labeling task, contributors are fairly accurate in detecting both positive and negative cells, resulting in higher image labeling accuracy.

### Crowdsourcing Performance

**Figure 6:**
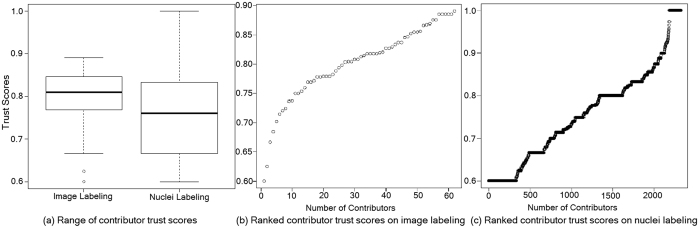
Contributor trust scores analysis on Crowdsourcing jobs.

We first assessed the contributor (crowd) performance for both crowdsourcing jobs. The number of contributors who participated in both jobs is shown in Table 2. The contributors who maintained the minimum accuracy (60%) on test questions during quiz and work modes are trusted contributors and the rest are untrusted contributors. In work mode, there were 61 trusted contributors for image labeling and 2,216 for nuclei labeling. The average time of trusted contributors was 32 seconds for image labeling and 306 seconds for nuclei labeling per image while the average time of untrusted contributors was 149 seconds for image labeling and 207 seconds for nuclei labeling. Thus, trusted contributors took less time to label images as compared to untrusted contributors; however, trusted contributors took more time to label nuclei as compared to untrusted contributors. These results suggest that efficient labeling of nuclei is a complex job requiring sufficient time for strong performance. Figure 6 illustrates the distribution of crowd trust scores for both jobs. The image labeling contributors have higher trust score as compared to nuclei labeling contributors. The average test question accuracy for trusted contributors is 80% for image labeling and 76% for nuclei labeling while average test question accuracy for untrusted contributors is 66% for image labeling and 42% for nuclei labeling. The trust scores were moderately correlated with the number of images labeled; *ρ* = 0.41, *P* < 0.0008 for image labeling job and *ρ* = 0.186, *P* < 2.2*e*^−16^ for nuclei labeling. The average time for image labeling is 50 seconds and nuclei labeling per image is 373 seconds.

The image labeling job was finished in 4 hours and nuclei labeling was finished in 472 hours. The Crowdflower platform charged $282 for image labeling and $2,280 for nuclei labeling job. These data suggest that although nuclei labeling produced some improvements in accuracy, it took longer (468 hours; 118-fold increase) and cost more ($1,998; ~8-fold increase) to complete the task.

**Figure 7:**
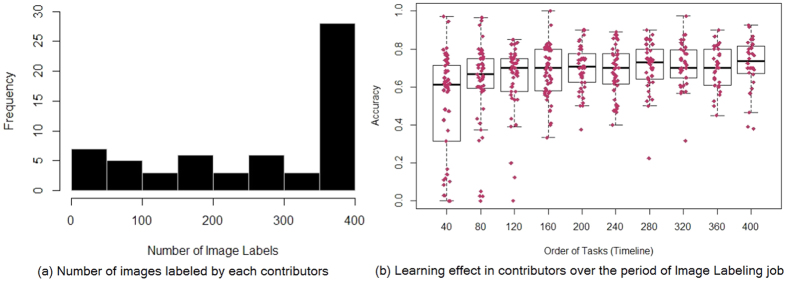
Contributor learning effect over the period of time on image labeling job.

We computed learning effect of contributors for image labeling. The numbers of images labeled by different contributors are reported in Fig. 7(a). For each contributor, we divide the image labeling time line into 10 different groups with the order of completions. Each group of image labeling time line consists of 40 images. Within each group, we compute the accuracy of each contributor for all images labeled during that time period. The learning curve of contributors during the order of job is reported in Fig. 7(b). In start of the job, more contributors have low accuracy, but they improved their accuracy over the time by learning. There was a positive correlation between the number of cases a crowd contributor had scored and their accuracy (spearman Rho = 0.23 and P-value = 1.83e-6) supporting that crowd members became more skilled as they scored more cases.

## Discussion

The principle of applying crowdsourcing in science, which has enabled whale sound classification^10^, malaria parasite classification^11^, sleep spindle detection^13^ and nuclei detection and segmentation in histopathology^15^, has become increasingly well established in recent years. Crowdsourced work can be used to classify objects (whale sound and malaria parasite classification), detect objects (nuclei detection) and segment objects (nuclei segmentation). The aim of this study was to better understand how to use crowdsourcing for IHC image interpretation. This laborious and time consuming image quantification task has also been performed using automated methods^4^,^5^,^6^,^20^,^21^,^22^. However, no prior studies have directly compared crowdsourcing vs. automated methods in the interpretation of IHC.

In this study, we quantify IHC TMA images for labeling of ER status with two different crowdsourcing approaches, image labeling and nuclei labeling. In the image labeling task, the crowd was asked to estimate the percentage of positive cells for each IHC image, while in nuclei labeling task, the crowd was asked to label individual nuclei within IHC images as either positive or negative. We completed these crowdsourcing tasks on a large data set containing 5338 TMA images belonging to 1853 patients, which were previously labeled by an expert pathologist and by an automated method. In our study, crowdsourcing-derived scores obtained greater concordance with the pathologist interpretation for both image labeling and nuclei labeling tasks, as compared with the pathologist concordance achieved by the automated method.

Directly comparing the results of our study with previously published crowdsourced IHC scoring publications is not straightforward due to differences in type of crowd sourcing, type of data sets used and evaluation metrics. Della Mea *et al*.^16^ crowdsourced only 13 IHC images for scoring of positivity index using nuclei labeling and reported 0.95 Spearman correlation. In another study using the CellSlider online application by Cancer Research UK^17^, researchers collected image labels for 6,378 breast TMA and reported overall 78% classification accuracy. In our study, we evaluated both crowdsourced based image labeling and nuclei labeling on same data set consisting of 5338 TMA cores from 1853 Patients. Furthermore, we also computed IHC scoring using Definiens Tissue Studio automated method (a commonly used software in research community for IHC scoring). Overall, the crowdsourced scores produced from nuclei labeling (as opposed to image labeling and Definiens) showed somewhat higher agreement with the pathologist scores; however, the time and cost required for the nuclei labeling far exceeded the time and cost for the image labeling.

Nuclei labeling is a more laborious task spread over many people, costs more and takes a longer time for nuclei scoring than image labeling. Our study results support that crowdsourcing is a promising new approach for scoring biomarker studies in large scale cancer molecular pathology studies. A limitation of our current crowdsourcing application is that we do not ask the Crowd to classify nuclei into specific types (e.g., cancer epithelial nucleus, lymphocyte nucleus). We expect the addition of training the crowd to classify cell types in addition to classifying IHC positivity will further improve crowd performance, although the incorporation of cell type-specific scoring may increase the time and cost of the overall task. This represents an important direction for future research.

## Additional Information

**How to cite this article**: Irshad, H. *et al*. Crowdsourcing scoring of immunohistochemistry images: Evaluating Performance of the Crowd and an Automated Computational Method. *Sci. Rep.* **7**, 43286; doi: 10.1038/srep43286 (2017).

**Publisher's note:** Springer Nature remains neutral with regard to jurisdictional claims in published maps and institutional affiliations.

## Figures and Tables

**Table 1:** Comparison of three methods (Definiens, crowdsourced image labeling and crowdsourced nuclei labeling) for IHC image classification.

Types	Methods	3-Class Labeling		2-Class Labeling	
		*A*_*g*_	*ρ*	*A*_*g*_	*ρ*
*Image Labeling	Crowd CV	**0.71**	0.64	**0.83**	**0.62**
	Crowd CT	0.68	**0.65**	0.81	0.61
	Crowd *ω*CV	0.64	0.63	0.77	0.59
	Crowd *ω*CT	0.64	0.64	0.77	0.59
*Nuclei Labeling	Crowd	**0.77**	**0.68**	**0.87**	**0.63**
	Definiens	0.70	0.51	0.81	0.48

Crowdsourced Nuclei labeling reported higher percentage agreement (*Ag*) and Spearman correlation (*ρ*) as compared with Definiens and Crowdsourced image labeling.

**Table 2:** Crowd performance on test questions in quiz mode and work mode.

Crowdsourcing Jobs	Quiz Mode		Work Mode	
	Passed	Failed	Passed	Failed
Image Labeling	113	155	61	52
Nuclei Labeling	3,244	1,572	2,216	1,243

**Table 3:** Confusion Matrix.

Image Labels	Crowd Image Labeling			Crowd Nuclei Labeling			Definiens Labeling			Actual
	Neg	Low Pos	Pos	Neg	Low Pos	Pos	Neg	Low Pos	Pos	
Neg	**151**	174	127	**235**	145	72	**113**	125	214	452
Low Pos	15	**47**	160	21	**50**	151	11	**37**	174	222
Pos	13	48	**1,118**	11	34	**1,134**	7	32	**1,140**	1,179
Predicted	179	269	1,405	267	229	1,357	113	194	1,528	1,853

Neg represents ER Negative, Low Pos represents ER Low Positive and Pos represents ER Positive.
